# Sigmoid Volvulus Following Mechanical Bowel Preparation in an Elderly Patient

**DOI:** 10.7759/cureus.106570

**Published:** 2026-04-07

**Authors:** Andy J Kim, Rebecca Reardon-Lochbaum, Matthew Kalliath

**Affiliations:** 1 Surgery, UMass Chan Medical School, Worcester, USA; 2 Surgery, Boston University Chobanian & Avedisian School of Medicine, Boston, USA; 3 Surgery, Cape Cod Hospital, Hyannis, USA

**Keywords:** colon resection, colon volvulus, hartmann procedure, intestinal perforation, perforated colon, pneumo peritoneum, sigmoid colectomy, sigmoid-end colostomy, sigmoid perforation, sigmoid volvulus

## Abstract

A male in his 80s presented with abdominal distension and altered mental status after receiving a polyethylene glycol-based mechanical bowel preparation prior to a diagnostic colonoscopy. He had a history of dementia, atrial fibrillation, and thrombocytopenia. Physical exam was significant for peritonitis, and computed tomography imaging revealed pneumoperitoneum. The patient underwent an exploratory laparotomy, where a perforated sigmoid volvulus was identified. Sigmoid colectomy with end colostomy was performed. The post-operative course was complicated by delirium and upper gastrointestinal bleeding due to stress gastritis. The patient was eventually discharged back to his prior assisted-living facility in stable condition. This case highlights that in patients with risk factors for sigmoid volvulus, such as anatomic abnormality, advanced age, and dementia, mechanical bowel preparation may act as a precipitating stressor. It underscores the need for careful assessment of risks and benefits when considering colonoscopy in older adults, including consideration of risk factors for volvulus and bowel perforation following mechanical bowel preparation.

## Introduction

Colonic volvulus is a condition in which the colon twists around its mesentery. Failure of the colon to untwist can cause bowel obstruction and intestinal ischemia. If untreated, this can progress to life-threatening complications such as necrosis and perforation [1]. Volvulus accounts for 10-15% of cases of large bowel obstruction in the United States [2]. Among the colonic segments, the sigmoid colon is the most commonly involved segment, accounting for 60-75% of cases [2]. The most common predisposing factor for sigmoid volvulus is anatomic abnormality, such as a redundant sigmoid colon with a narrow mesenteric base. Advanced age is also an established risk factor, but age-stratified data are limited, and available literature reports incidence in aggregate populations. While the etiology of colon redundancy is debated, chronic constipation is a leading theory, particularly in elderly patients and those with neuropsychiatric disorders that impair gastrointestinal motility [1,3].

Initial workup of sigmoid volvulus should begin with a focused history, likely notable for an acute onset of bloating, abdominal discomfort, reduced tolerance of oral intake, and obstipation, and physical examination, which may reveal abdominal distension, tympany, and an empty rectal ampulla. Signs of peritonitis may be present in volvulus complicated by bowel necrosis or perforation. Laboratory tests and imaging are essential in the workup. Leukocytosis is a common finding. Elevated lactate may be found in complicated volvulus. An abdominal radiograph may show a classic “coffee bean sign.” An abdominal/pelvic CT scan is considered the gold standard imaging study and may be considered when there is concern for necrosis or perforation. This will often demonstrate a dilated colon and a “whirl sign,” which suggests twisting of mesenteric vessels. Although uncomplicated volvulus can be treated with endoscopic decompression and detorsion followed by elective surgery to prevent recurrence, complicated volvulus requires emergency surgery [2].

A study on 19,220 sigmoid volvulus cases in the United States indicated an overall mortality of 9.4% in patients who underwent colon resection [4]. The most common post-operative complications included ileus/bowel obstruction, urinary tract infection, and anastomotic complications such as leak, fistula, and abscess. The strongest predictor of mortality was complicated volvulus, as evidenced by signs of peritonitis, bowel gangrene, and/or necrosis.

## Case presentation

**Figure 1:**
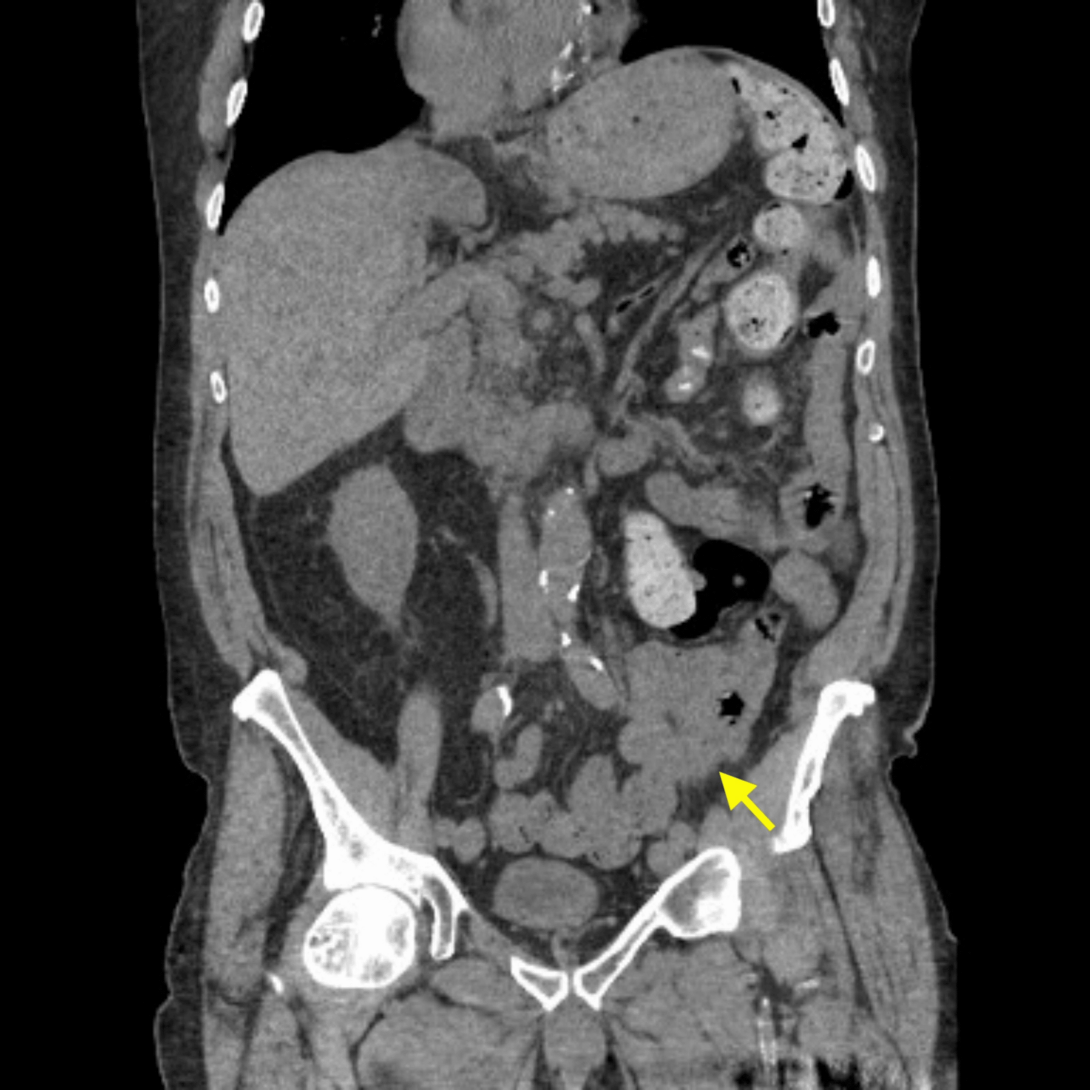
Abdominal/pelvic CT without contrast, coronal section demonstrating redundancy of sigmoid colon with dilatation up to 9 cm, taken three years prior to admission CT, Computed Tomography

A male in his 80s presented to the emergency department from an assisted-living facility with one day of abdominal distension and altered mental status after undergoing mechanical bowel preparation with polyethylene glycol-electrolyte solution in anticipation of a diagnostic colonoscopy. Past medical history was significant for dementia, atrial fibrillation on apixaban, gastroesophageal reflux disease, anemia, thrombocytopenia, and prostate cancer. Notably, an abdominal/pelvic CT scan performed three years prior for evaluation of hematuria revealed a dilated, redundant sigmoid colon (Figure 1).

On interview, the patient was acutely confused and disoriented. On exam, his abdomen was markedly distended and tympanitic with diffuse tenderness, concerning for peritonitis. He was afebrile, hemodynamically stable, and in rate-controlled atrial fibrillation. Laboratory tests revealed leukocytosis with white blood cells at 20.5 x 10^3^/mm^3^ (Table 1). He also had mild anemia and thrombocytopenia with a hemoglobin of 13.6 g/dL and platelet count of 98 x 10^3^/mm^3^, which were consistent with his baseline values. Other significant laboratory findings included hypokalemia, elevated blood urea nitrogen and creatinine, and mild mixed hyperbilirubinemia. The leukocytosis was consistent with systemic inflammatory response in the setting of bowel perforation. The elevated blood urea nitrogen and creatinine reflected pre-renal azotemia from dehydration, which may have been worsened by bowel preparation. The hypokalemia was likely multi-factorial, due to gastrointestinal losses during bowel preparation and renal compensation in the setting of dehydration. Mild mixed hyperbilirubinemia in this context may have represented an acute stress response or cholestasis from systemic illness rather than primary hepatobiliary pathology.

**Table 1:** Laboratory values on emergency department admission

	Reference Ranges and Units	Laboratory Values
Complete Blood Count		
Hemoglobin	13.7-17.5 g/dL	13.6
Hematocrit	40.1-51.0 %	40.7
White Blood Cells	4.2-9.1 10^3^/mm^3^	20.5
Platelets	163-337 10^3^/mm^3^	98
Chemical Profile		
Sodium	136-145 mEq/L	141
Potassium	3.5-5.1 mEq/L	3.0
Chloride	98-107 mEq/L	104
Bicarbonate	20-31 mEq/L	24
Blood Urea Nitrogen	9-23 mg/dL	34
Creatinine	0.73-1.18 mg/dL	1.38
Bilirubin, Total	0.2-1.1 mg/dL	2.4
Bilirubin, Direct	<=0.3 mg/dL	0.9
Bilirubin, Indirect	0.0-1.0 mg/dL	1.5

**Figure 2:**
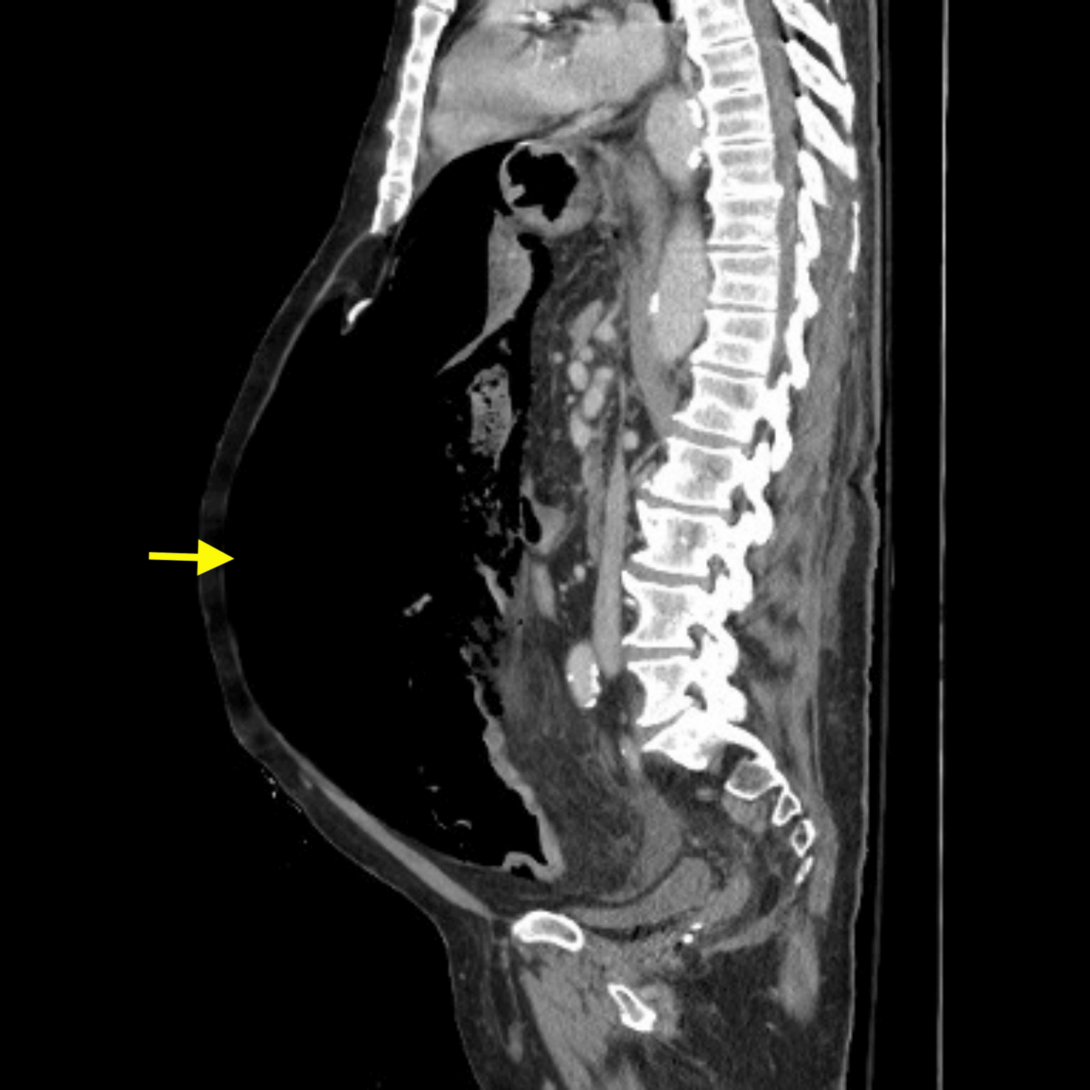
Abdominal/pelvic CT with intravenous contrast, sagittal section demonstrating pneumoperitoneum CT, Computed Tomography

**Figure 3:**
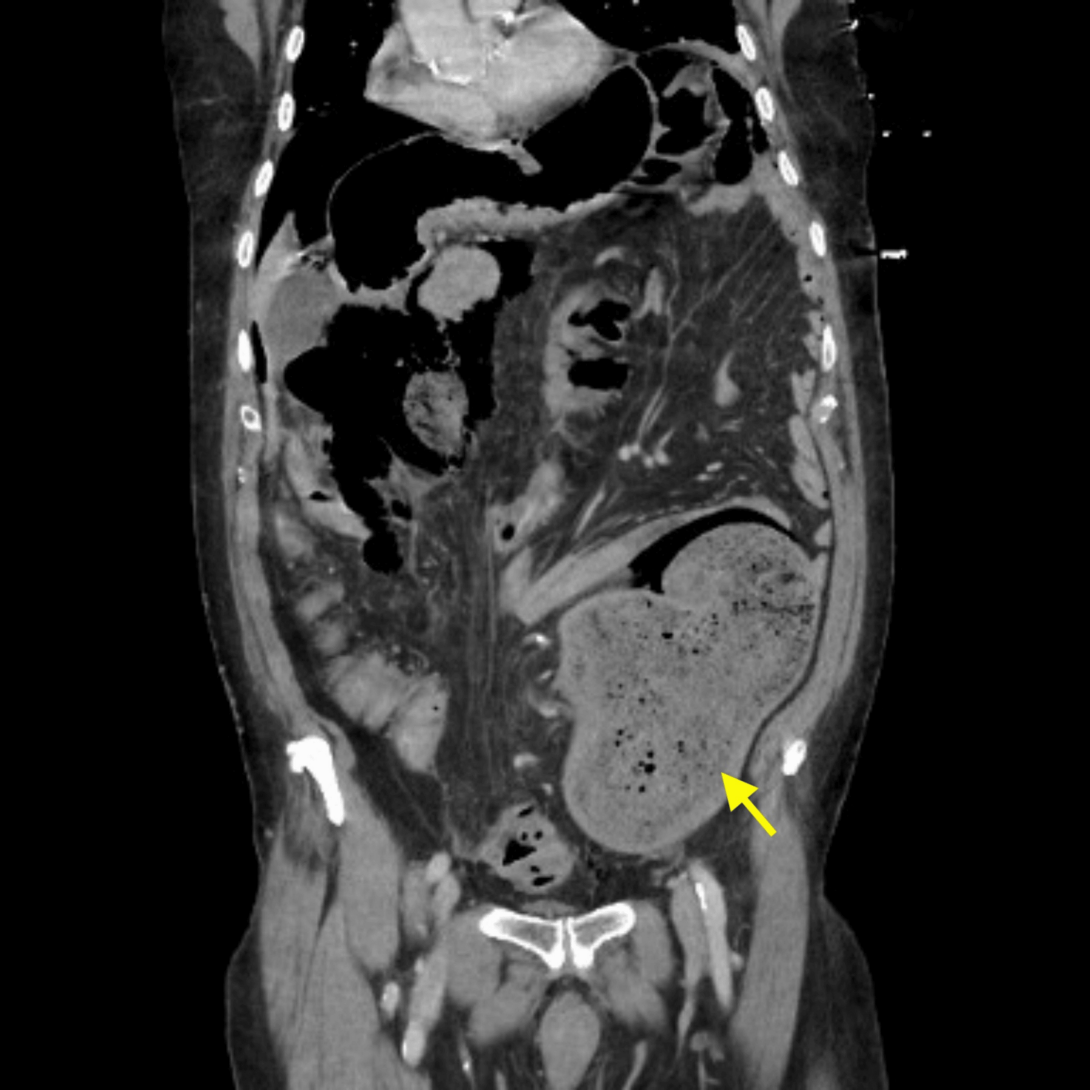
Abdominal/pelvic CT with intravenous contrast, coronal section demonstrating dilatation of the distal descending colon up to 10.5 cm that tapers in the mid-sigmoid region, concerning for sigmoid volvulus CT, Computed Tomography

An abdominal/pelvic CT with intravenous contrast demonstrated pneumoperitoneum (Figure 2) and massive distension of the distal descending and proximal sigmoid colon with sudden tapering at the middle sigmoid colon (Figure 3). These radiologic findings, in addition to the physical exam and abnormal laboratory findings, were most concerning for perforated sigmoid volvulus.

The decision was made to proceed with emergency surgery. The patient received intravenous fluid resuscitation and was started on broad-spectrum antibiotics for intraabdominal contamination. He underwent an exploratory laparotomy. Upon entry to the abdomen, the presumptive diagnosis of sigmoid volvulus was confirmed. The segment was detorsed, demonstrating a redundant, patulous sigmoid colon. There was diffuse emphysema of the mesentery, and a small area of transmural necrosis was identified. There was a large volume of liquid stool proximal to the obstruction. The decision was made to perform a resection of the diseased segment and create an end colostomy, or a Hartmann’s procedure. Final surgical pathology identified a large area of transmural hemorrhagic necrosis and diffuse submucosal edema, but no evidence of malignancy.

Overall, the patient demonstrated a favorable post-operative course. He was restarted on his home anticoagulation on post-operative day 5 and was continued on broad-spectrum antibiotics until post-operative day 8. He did develop hospital-related delirium but returned to his cognitive baseline prior to discharge. Discharge was further delayed due to acute blood loss secondary to an upper gastrointestinal hemorrhage on post-operative day 6, which necessitated upper endoscopy to obtain hemostasis of a bleeding ulcer at the gastroesophageal junction. He was discharged on post-operative day 10 to a short period of subacute rehabilitation before returning to his assisted-living facility.

At outpatient follow-up two weeks after discharge, the patient was doing well and had no further upper gastrointestinal bleeding. Two months post-operatively, he began to experience reducible stoma prolapse; discussions were had with the patient and his family regarding possible surgical intervention versus conservative measures. They opted for a watchful waiting approach.

## Discussion

In summary, this is a case of a male in his 80s who developed perforated sigmoid volvulus following mechanical bowel preparation in anticipation of a diagnostic colonoscopy. The patient successfully underwent an emergency Hartmann’s procedure. Prior to the operation, we discussed the possibility of sigmoid colectomy with primary anastomosis for this patient. Studies have not indicated significant differences in mortality and morbidity between a Hartmann’s procedure and colectomy with primary anastomosis [5,6]. However, in the setting of the patient’s baseline dementia that required assistance with activities of daily living in an assisted-living facility, we opted for a colostomy for perceived benefit to quality of life. The decision was made collaboratively with the patient’s power of attorney.

Our patient had several risk factors for developing sigmoid volvulus including redundant sigmoid colon, dementia, age greater than 65, and male sex. Given the temporal association, the mechanical bowel preparation may have contributed to the development of volvulus in a patient with multiple pre-existing risk factors. The possible association between mechanical bowel preparation and volvulus formation is further supported by the significant liquid stool burden proximal to the obstruction that was found intra-operatively. To our knowledge, this is the first published case report of complicated sigmoid volvulus after mechanical bowel preparation.

This report is limited by its nature as a single case, and therefore, a causal relationship between mechanical bowel preparation and sigmoid volvulus cannot be definitively established by this report alone. It is possible that bowel preparation served as a precipitating factor rather than the sole cause of volvulus. The patient had several well-established predisposing factors that may have independently increased his risk. Additionally, the temporal association between bowel preparation and symptom onset may represent coincidental timing rather than a true causal mechanism. Nevertheless, this case highlights a plausible association and a potentially underrecognized risk in vulnerable patient populations.

The development of bowel obstruction following mechanical bowel preparation has been noted as a rare complication that occurs in less than 1% of bowel preparations [7]. However, there have not been studies that explore the occurrence of this complication among increased-risk patients. Latos et al. reported 14 cases of partial obstruction that were resolved with conservative treatment and four cases of complete obstruction that required surgery in a study population of 9,962 bowel regimens, but this study did not stratify outcomes by patient risk factors and may underestimate complication rates in patients with predisposing risk factors for obstruction such as in our case.

In elderly patients, mechanical bowel preparation warrants additional caution. First, inadequate bowel preparation has been cited more often in elderly patients with comorbidities such as dementia and Parkinson’s disease, limiting the diagnostic utility of such preparation without mitigation of any risks it carries [8]. Second, some agents such as magnesium citrate have been advised against use in elderly patients or patients with renal insufficiency. Polyethylene glycol-electrolyte solution, as used in our case patient, is considered one of the most popular bowel preparation agents given its isosmotic property that minimizes electrolyte shifts. Abdominal side effects reported with the use of polyethylene glycol-electrolyte solution include nausea, vomiting, abdominal pain, and colitis.

Colon cancer screening is recommended for average-risk adults aged 45-75 with one of the following methods: (i) screening colonoscopy every 10 years, (ii) sigmoidoscopy every five years, or (iii) stool-based tests every 1-3 years [9,10]. For patients aged 75-85, it is unclear as to whether there is a benefit to routine screening. Many organizations recommend an individualized approach in this age range that considers the individual patient’s overall health and screening history. Screening colonoscopy is not advised for individuals aged 86 or older, and, in fact, the rate of mechanical complications following screening colonoscopy is higher in elderly patients with an associated increase in morbidity [11]. Stool-based immunochemical tests, which are less invasive screening tests that detect blood in stool, may provide a theoretical benefit in avoiding such mechanical complications, but there is limited evidence that demonstrates a mortality benefit from using these tests in this age group.

Given the morbidity and mortality associated with perforated sigmoid volvulus, further research is warranted to better define the risk-benefit profile of alternative diagnostic modalities such as sigmoidoscopy or fecal immunochemical test in these increased-risk patients. For patients over the age of 75, for whom recommendations for colon cancer screening are not definitive, risk factors for sigmoid volvulus should certainly be considered when choosing to pursue screening colonoscopy.

Diagnostic colonoscopy is an important tool to investigate suspicion for lower gastrointestinal pathology such as colon cancer. Common indications for a diagnostic colonoscopy include iron deficiency anemia, unintentional weight loss, and concerning lower gastrointestinal symptoms such as a change in bowel habits or hematochezia [12]. In this patient, the decision to pursue diagnostic colonoscopy was made based on his overall clinical context, given his frailty, unexplained thrombocytopenia, and lack of prior endoscopic evaluation for occult gastrointestinal pathology. While isolated thrombocytopenia alone is not a standard indication for colonoscopy, it contributed to the broader clinical picture that prompted diagnostic evaluation. This highlights how, in practice, the approach to diagnostic colonoscopy is often patient-specific, driven by individual factors rather than dictated solely by guidelines. In such scenarios where the indication for diagnostic colonoscopy is not clearly delineated by guidelines, consideration of patient-specific risk factors for complications, such as colonic redundancy, may help guide decision-making. 

## Conclusions

This case highlights a rare but serious complication occurring in temporal association with mechanical bowel preparation in an elderly patient with multiple predisposing risk factors for sigmoid volvulus. Mechanical bowel preparation may act as a precipitating stressor in individuals with underlying colonic anatomic abnormalities or other risk factors for sigmoid volvulus. Further investigation is warranted to clarify the association of mechanical bowel preparation with sigmoid volvulus. Clinicians should consider this potential association when assessing the risks and benefits associated with mechanical bowel preparation for colonoscopy in older adults.
